# Supplementary material for: WordCommentsAnalyzer: A windows software tool for qualitative research
Source: F1000Res. 2018 Sep 4;7:536. Originally published 2018 May 3. [Version 2] doi: 10.12688/f1000research.14819.2 (PMC6137411; doi:10.12688/f1000research.14819.2)
Supplement: Supplementary file 3 [file f1000research-7-17614-s0002.tgz › b2a79496-4ada-47c4-9682-a99ce867836d.zip!/Eight Iranian Health Journals/Journal of Research in Rehabilitation Sciences _19.docx]

© Journal of Research in Rehabilitation Sciences . This work is licensed under CC BY-NC 4.0 (https://creativecommons.org/licenses/by-nc/4.0/).

Volume & Issue: دوره 10، شماره 6، 1393

Title: بررسی تاثیر کفش های غلتکی و تمرینات اکستانسوری مکنزی بر میزان درد، ناتوانی و پوسچر کمر در افراد مبتلا به کمردرد تحت حاد

http://jrrs.mui.ac.ir/index.php/jrrs/article/view/2047

چکیده مقدمه: کمردرد یکی از شایع ترین اختلالات اسکلتی-عضلانی می باشد. اگرچه اغلب تمرین درمانی توصیه  می شود اما نیازمند زمان ، تجهیزات و در خطر رعایت نکردن تمرینات از طرف بیمار است. کفش های غلتکی با تغییراتی در پوسچر مچ پا ، زانو ، ران و کمر می توانند به عنوان بخشی از درمان  استفاده شوند.  بنابراین هدف از این مطالعه بررسی تاثیر کفش های غلتکی و تمرینات اکستانسوری مکنزی بر میزان درد، ناتوانی و پوسچر کمر در افراد مبتلا به کمردرد تحت حاد می باشد. مواد و روش ها: در مطالعه شبه تجربی حاضر، با روش نمونه گیری در دسترس (convenience) 20 فرد ( 10 مرد و 10 زن ) با میانگین سن  45/10± 15/32  مبتلا به کمردرد تحت حاد که با انجام حرکات تکراری اکستانسیون در حالت ایستاده و دمر(پرون) درد آن ها کاهش می یافت ، پس از امضا کردن فرم رضایتنامه آگاهانه در دو گروه تمرینات اکستانسوری مکنزی(10n=) و گروه تمرینات اکستانسوری مکنزی و کفش غلتکی(10n=) قرار گرفتند. قبل و بعد از 4 هفته مداخلات درمانی، شدت درد (معیار دیداری درد )، ناتوانی (پرسشنامه Oswestry) و پوسچر کمر (دستگاه Qualisys Track Manager  مجهز به 7 دوربین 100Hz  ورژن 2.10 ،ساخته و طراحی شده توسط کارخانه  Qualisys کشور سوئد)  اندازه گیری شد. یافته ها: میزان کاهش درد در گروه تمرینات اکستانسوری و کفش به طور معنادار و به میزان 9/58٪ بیشتر از گروه تمرینات اکستانسوری بود (04/0P=). کاهش میزان ناتوانی، در مقایسه بین دو گروه از نظر آماری معنادار نبود (05/0P>). شعاع قوس کمر در هر دو گروه تغییر نکرد (05/0P>) و همچنین اختلاف معنادار در شعاع قوس کمر بین دو گروه وجود نداشت (05/0P>). نتیجه گیری: نتایج نشان میدهد کفش های غلتکی می توانند به عنوان بخشی از برنامه درمانی افراد مبتلا به کمردرد تحت حاد باشد.

Title: بررسي تاثیر برنامه كاردرماني خانواده محور بر بهبود مهارت¬هاي حركتي كودكان مبتلا به فلج مغزي اسپاستیک

http://jrrs.mui.ac.ir/index.php/jrrs/article/view/1699

چکیده فارسی مقدمه: فلج مغزی شایع ترین ناتوانی حرکتی مزمن و عارضه عصب- شناختی در کودکان است. همکاری فعالانه والدین در اجرای برنامه­های توانبخشی کودک، رشد و تکامل حرکتی کودک را تسهیل خواهد کرد. در پژوهش حاضر به تعيين اثربخشي برنامه كاردرماني خانواده محور بر توسعه مهارت­هاي حركتي كودكان فلج مغزي اسپاستيك پرداخته شد. مواد و روش­ها: در این مطالعه مداخله­ای و شبه تجربی، 20 كودك فلج مغزي اسپاستیک 7-5 ساله كه توانایی راه رفتن مستقل داشتند از جامعه در دسترس انتخاب شده و به صورت تصادفي در دو گروه 10 نفره مداخله و گواه قرار گرفتند. گروه مداخله علاوه بر درمان­های روتین توانبخشی، برنامه کاردرمانی30 جلسه­ای خانواده محور را نیز دریافت نمودند. قبل و پس از اجرای برنامه توسط والدین هر دو گروه آزمایش و گواه با استفاده از آزمون کفایت حرکتی برونینکز- اوزرتسکی (BOTMP) مورد ارزیابی قرار گرفتند و داده­های جمع آوری شده با استفاده از تحلیل کوواریانس تک متغیره مورد بررسی قرار گرفت. یافته­ها: نتایج نشان می­دهد که گروه آزمایش پس از شرکت در برنامه مداخله، در مقایسه با گروه کنترل، نه تنها از لحاظ آماری تفاوت معناداری را نشان داده است (001/0 = P) بلکه اندازه اثر بدست آمده (62/0=ηP2) نشان دهنده­ی اثر بخشی مداخله بر روی مهارت­های حرکتی کودکان فلج مغزی در آزمون کفایت حرکتی برونینکز- اوزرتسکی است. نتیجه گیری: برنامه­ي كاردرماني خانواده محور در كنار برنامه­هاي متداول توان بخشي، مي تواند نقش مهمی در بهبود و توسعه­ي مهارت­هاي حركتي كودكان فلج مغزي داشته باشد.

Title: دیرش انتقال فرمنت دوم در گفتار روان افراد مبتلا به لکنت

http://jrrs.mui.ac.ir/index.php/jrrs/article/view/2086

چکیده: مقدمه: نتایج بسیاری از مطالعات روی جنبه‌های حرکتی و صوتی افراد مبتلا به لکنت نشان می دهد که گفتار  روانِ افراد مبتلا به لکنت با گفتار افراد بدون لکنت متفاوت است. یکی از این جنبه‌های رمزگشایی و پردازشی که در مطالعات مورد مطالعه قرار گرفتند، ویژگی های انتقال فرمنت واکه‌ای است. با توجه به اینکه ساختار فرمنتی از جنبة زمانی و فرکانسی نشان دهندة پویایی سیستم تولیدی به خصوص زبان است می تواند در بررسی توانایی های حرکتی افراد مبتلا به لکنت مورد استفاده قرار گیرد. هدف از این مطالعه بررسی دیرش انتقال فرمنت دوم در گفتار روان افراد مبتلا به لکنت و مقایسة آن با افراد بدون لکنت است. مواد و روش ها: این مطالعه از نوع تحلیلی - مقطعی  می‌باشد. تعداد 20 نفر مبتلا به لکنت و 20 نفر از افراد طبیعی انتخاب شدند و 9 نمونه ی گفتاری در قالب یک عبارت حامل ارائه شده و گفته ها ضبط شدند. داده های صوتی با استفاده از نرم افزار Praat تحلیل شده و زمان دیرش انتقال فرمنت دوم بر حسب میلی ثانیه به دست آمدند. یافته ها: طبق یافته های این پژوهش دیرش انتقال فرمنت دوم در گروه افراد مبتلا به لکنت کمتر از افراد بدون لکنت  بود در حالیکه این تفاوت از لحاظ آماری معنادار نبود. نتیجه گیری: با توجه به نتایج این مطالعه طبق معیار دیرش انتقال فرمنت دوم می توان گفت توانایی های پردازش حرکتی گفتار از جنبه زمانی در افراد لکنتی با افراد بدون لکنت متفاوت است و این افراد در حرکت از موقعیت یک همخوان به واکه بعدی سریعتر عمل می کنند.

Title: مقايسه استفاده از زمان، اوقات فراغت و کیفیت مدیریت زمان در مادران کودکان مبتلا به فلج‌مغزی با مادران كودكان سالم در شهر اصفهان

http://jrrs.mui.ac.ir/index.php/jrrs/article/view/2010

چکیده:          مقدمه: مراقبت از کودک با فلج مغزی وقت و انرژی زیادی را از مادر میگیرد همین امر باعث شده مادران زمان کافی برای کارهای دیگر اختصاص ندهند، لذا در این پژوهش الگوی استفاده از زمان مادران کودکان با فلج مغزی مورد بررسی قرار گرفته است. موارد و روش‌ها: این مطالعه از نوع مورد- شاهد انتخاب و در شهر اصفهان انجام شد. در گروه مادران با کودکان سالم30 نفر و در گروه مادران کودکان با فلج مغزی نیز 30 نفر شرکت کننده وجود داشت. با استفاده از پرسش نامه "استفاده از زمان مادران"  و نيز پرسش نامه دموگرافيك داده هاي لازم براي پژوهش جمع آوري شد. از آزمون های Mann–Whitney و Independent t برای تحلیل داده ها در SPSS16  استفاده شد.  نتایج: آنالیز آماری داده ها تفاوت معنی داری را در حیطه مراقبت از کودک نشان داد به طوری که مادران کودکان با فلج مغزی زمان بیشتری را صرف امور کودک میکردند. در میزان رضایت از مهارت مدیریت زمان نیز تفاوت معنی داری به دست آمد و مادران داری کودکان سالم رضایت بیشتری داشتند. بحث: بر اساس اطلاعات به دست آمده از این پژوهش، مادران کودکان با فلج مغزی زمان بیشتری را صرف امور کودک میکنند درنتیجه برای کارهای دیگر همچون مراقبت از خود و خواب/استراحت زمان کمتری را اختصاص داده و همین امر منجر به رضایت کمتر از نحوه ی استفاده از زمان در این مادران میگردد.

Title: عنوان: اثر تخت ترکشن بر ستون فقرات ناحیه کمری با روش اجزای محدود و روش مدلسازی

http://jrrs.mui.ac.ir/index.php/jrrs/article/view/2000

مقدمه:کمردردیکی از مهمترین مسائلی است که در سرتاسر جهان وجود دارد. حدود 80 تا 90% کمردرد ها از نوع حاد است که فتق دیسک یا Herniated Discدر بین مهره‌های L4-L5 ناحیه کمری و پارگی دیسک از مهمترین دلایل این نوع کمردرد هستند. روش درمانی که برای این نوع کمردرد استفاده می‌شود، کشش ستون فقرات با تخت ترکشن است. تخت ترکشن با افزایش ارتفاع دیسک‌ها و ایجاد فاصله بین مفاصل فاست به درمان کمردرد کمک می‌کند. مواد و روش‌ها: در این مطالعه، با استفاده از تصاویر سی‌تی‌اسکن گرفته‌ شده از یک بیمار زن 31 ساله به وزن 83 کیلوگرم، مدل سه بعدی ستون فقرات در ناحیه کمری با استفاده از نرم‌افزار Mimics10.01 ساخته‌ شد و مدل جهت تحلیل وارد نرم‌افزار Abaqus6.11 شد و در این نرم‌افزار خواص و ویژگی‌های اجزای ستون فقرات مدل شد و بارگذاری شبیه بارگذاری تخت ترکشن برروی مدل انجام شد و سپس تحلیل نتایج بدست آمده با مطالعات تجربی گذشته مورد بررسی قرار گرقت. هدف از انجام این تحقیق ارائه یک روش برای بررسی میزان اثر بخشی تخت ترکشن بر ستون فقرات بود و این روش توسط مدلسازی سه بعدی و استفاده از روش اجزای محدود ارائه شد. نتایج: پس از انجام بارگذاری در نرم‌افزار آباکوس، میزان جابجایی دیسک‌ها در بین مهره‌های L4-L5 و L3-L4 مورد بررسیقرار گرفت که این جابجایی‌ها به ترتیب برابر 59/2 میلی‌متر و 81/1 میلی‌متر بود. این نتایج با نتایج حاصل از بررسی‌های آزمایشگاهی، که از طریق بررسی جابجایی دیسک‌ها توسط عکس‌های ام‌آرآی و سی‌تی‌اسکن قبل و بعد از ترکشن بدست‌آمده بودند، مقایسه شد و مشخص شد که نتایج تطابق قابل قبولی با همدیگر دارند. بحث: تخت ترکشن اثربخشی مناسبی در جابجایی دیسک‌ها و کاهش درد در ستون فقرات دارد.کلید‌ واژه- تخت ترکشن، مدل‌سازی، کمردرد، اجزای محدود

Title: تأثیر تمرینات اصلاحی بر کیفوزیس و تعادل نوجوانان کم توانی ذهنی آموزش پذیر

http://jrrs.mui.ac.ir/index.php/jrrs/article/view/1712

چکیده: مقدمه: افراد با کم‌توانی ذهنی به دلیل نگرش غلطی که در جامعه وجود دارد جز افراد کم‌تحرک محسوب می­شوند. عدم وجود تحرک مناسب و اتخاذ وضعیت غلط بدنی می­تواند باعث ایجاد ناهنجاری کیفوزیس­ پشتی و در پی آن کاهش تعادل گردد. هدف این مطالعه، تعیین تأثیر شش هفته تمرینات اصلاحی بر میزان درجه کیفوزیس و تعادل افراد با کم‌توانی ذهنی آموزش پذیر بود. مواد و روش­ها: در این مطالعه شبه تجربی، تعداد 30 نفر کم‌توان ذهنی نوجوان دچار کیفوزیس با توجه به بهره هوشی در دو گروه کنترل و تجربی (هر گروه 15 نفر) تقسیم شدند. گروه کنترل به زندگی روزمره خود ادامه داد درحالی‌که گروه تجربی به مدت شش هفته و به‌صورت سه جلسه 45 دقیقه­ای در هفته به تمرینات اصلاحی پرداختند. میزان درجه کیفوزیس، تعادل پویا و تعادل ایستا قبل و بعد از تمرینات اصلاحی به‌وسیله خط­کش منعطف، آزمون راه رفتن پاشنه به پنجه و ایستادن روی دستگاه فوت اسکن به مدت 25 ثانیه اندازه­گیری شد. داده­های آماری با آزمون tمستقل تجزیه‌وتحلیل شدند ( 05/0≥p). یافته­ها:نتایج نشان داد که تمرینات اصلاحی باعث کاهش میزان درجه کایفوزیس (001/0≥p)، افزایش تعادل ایستا (001/0≥p) و افزایش تعادل پویا (001/0≥p) در گروه تجربی شد. در گروه کنترل تغییرات معنی­داری مشاهده نشد. نتیجه­گیری: با توجه به نتایج این مطالعه، تمرینات اصلاحی می‌تواند باعث کاهش میزان درجه کایفوزیس و افزایش تعادل در افراد با کم‌توانی ذهنی شود. این نتایج حاکی از نیاز بیشتر افراد کم‌توان ذهنی به تحرک و تمرینات ورزشی است.

Title: تاثیر آموزش گروهی بر دانش و نگرش مراقبین از کودک کم توان ذهنی

http://jrrs.mui.ac.ir/index.php/jrrs/article/view/1826

چکی مقدمه: داشتن کودک کم توان ذهنی مشکلات جسمی، روحی، روانی و اجتماعی زیادی را بر واحد خانواده تحمیل می‌کند و آنان را دچار آسیب پذیری می‌نماید. انجام مداخلات مناسب برای بهبود دانش و ایجاد نگرش مطلوب می‌تواند تا حد زیادی حامی این خانواده‌ها بوده و کیفیت زندگی بهتری را به آنان هدیه نماید. این مطالعه با هدف تعیین تاثیر آموزش گروهی بر دانش و نگرش مراقبین از کودک کم توان ذهنی انجام شده است. مواد و روش‌ها: این مطالعه از نوع مداخله‌ای تصادفی کنترل‌دار می‌باشد که در سال1392 در مرکز توانبخشی نرگس میانه انجام گرفت. 64 مراقب دارای کودک کم توان ذهنی 18-6 ساله بطور تصادفی به دو گروه مداخله و کنترل تقسیم شدند. برای گروه مداخله 6 جلسه کلاس آموزشی برگزار گردید ولی برای گروه کنترل هیچ مداخله‌ای انجام نشد. در هر دو گروه در ابتدا و انتهای مطالعه، داده‌ها براساس پرسشنامه‌های محقق ساخته دانش و نگرش جمع آوری گردید. داده‌ها از طریق نرم افزار  SPSS21و با استفاده از آزمون‌های آماری تی زوج، تی مستقل و کای اسکوئر  مورد تجزیه و تحلیل قرار گرفت. یافته ها:بین میانگین دانش، نگرش و رفتار مراقبین در هر دو گروه، قبل از مداخله اختلاف آماری معنی داری بدست نیامد. نتایج نشان داد 5/1 ماه بعد از اجرای مداخله، افزایش میزان دانش و نگرش مراقبین در گروه مداخله قبل(80/2±54/8، 51/6±29/62) و بعد از مداخله(86/1±50/14، 76/3±57/72) معنی دار بود(05/0P<). نتیجه گیری: آموزش گروهی به مراقبین از کودکان کم توان ذهنی، باعث افزایش دانش و نگرش آنان می‌گردد. توجه به افزایش آگاهی، درک و نگرش مراقبت کننده، می تواند وی را در امر مراقبت تخصصی و کارآمد یاری دهد.

Title: بررسی همبستگی بین آزمون های تعادل و تحرک عملکردی و پارامترهای نوسان مرکز فشار در حالت ایستاده آرام با و بدون تکلیف شناختی در کودکان با فلج مغزی همی پارزی

http://jrrs.mui.ac.ir/index.php/jrrs/article/view/1943

مقدمه: به دلیل تاثیر شخص، تکلیف و محیط بر روی کنترل وضعیتی کودکان با فلج مغزی همی پارزی، روش های مختلف بالینی و آزمایشگاهی برای سنجش کنترل وضعیتی مطرح است. هدف از این مطالعه بررسی همبستگی میان آزمون های بالینی و آزمایشگاهی تعادل در کودکان با فلج مغزی همی پارزی بود. مواد و روش ها: در این مطالعه همبستگی، 28 کودک فلج مغزی همی پارزی با متوسط سنی(34/2±) 09/10  سال به روش نمونه گیری غیر احتمالی ساده انتخاب شدند. آزمون های TUG)) Timed Up & Go، (FR) Functional Reach، Bruininks Oseretsky Test of Motor Proficiency (BOTMP)   و پارامترهای سرعت متوسط، طول مسیر، صفحه فاز کل و سطح کل نوسانات مرکز فشار در سه سطح سختی وضعیتی (سطح سفت با چشم باز و بسته و سطح فوم با چشم بسته در حالت ایستاده آرام) و دو سطح سختی شناختی (با و بدون تکلیف شناختی) ثبت شده با صفحه نیرو به ترتیب به عنوان آزمون های بالینی و آزمایشگاهی استفاده شد. نتایج: از میان 3 تکلیف وضعیتی فقط در ایستادن روی فوم با چشم بسته همبستگی متوسط تا بسیار بالایی بین تمامی پارامترهای نوسان مرکز فشار و آزمون های TUG، FR و BOTMP دیده شد. تکلیف شناختی در حالت ایستادن روی سطح فوم با چشم بسته باعث افزایش همبستگی بین آزمون های بالینی و آزمایشگاهی گردید. بحث: همبستگی مناسبی بین آزمون های تعادل و تحرک عملکردی و پارامترهای نوسان مرکز فشار در حالت ایستادن روی سطح فوم با چشم بسته به دست آمد.

Title: تعامل بین اعداد و توجه فضایی در افراد ایرانی

http://jrrs.mui.ac.ir/index.php/jrrs/article/view/1989

چکیده مقدمه  مطالعات قبلی بر روی بازنمایی ذهنی اعداد حاکی از وجود رابطه بین اعداد و فضا می باشد. از این بازنمایی به عنوان محور ذهنی اعداد یاد می­شود که در آن اعداد کوچک در سمت چپ و اعداد بزرگ در سمت چپ می باشند. همچنین معلوم شده است که این بازنمایی تحت تأثیر جهت خط نوشتاری قرار دارد. هدف این مطالعه بررسی رابطه عدد- فضا و تعیین خاستگاه آن با توجه به ویژگی­های خط فارسی بود. روش در تجربه اول 30 دانشجو برای تکمیل دو تکلیف نمادین و غیر نمادین توجه پوسنر مشارکت کردند. در تجربه دوم نیز همان تکلیف نمادین برای 37 نفر دانشجوی دیگر ارائه شد و پتانسیل­های برانگیخته وابسته به رویداد توسط دستگاه الکتروآنسفالوگرافی ثبت گردید. نتایج داد­ه­ها توسط آزمون آماری تکرار سنجش (RANOVA) مورد بررسی قرار گرفت. تعامل معناداری بین بزرگی و سمت محرک نبود (51/0p= ,437/0F=). اثر بزرگی معنادار بود (03/0p= ,21/5F=). بنحوی که سرعت پاسخ دهی کمیت­های بزرگتر (14±475 هزارم ثانیه)، بیشتر از سرعت پاسخ دهی برای کمیت­های کوچکتر (13±468 هزارم ثانیه) بود. همچنین سرعت پاسخ­دهی برای کمیت­های غیر نمادین کندتر (14±479) از کمیت­های ­نمادین (13±464) بود (03/0p= ,12/5F=). نتایج تجربه دوم نیز مشابه تجربه اول بود یعنی تنها اثر اصلی بزرگی معنادار بود (005/0p= ,612/9F=). هیچ کدام از اثرات اصلی و تعاملی نهفتگی مؤلفه P300 نبود. نتیجه­گیری نتایج رفتاری حاکی از وجود اثر اندازه بود. لیکن تعامل بین بزرگی و سمت محرک هدف (پدیده SNARC) مشاهده نشد. یعنی مشاهده بزرگی باعث سوق توجه ذهنی به سمت راست یا چپ نگردید. نتایج ERP نیز نتایج رفتاری را تائید کرد. بطور خلاصه، پدیده تعامل بزرگی اعداد با سمت ارائه محرک در ایرانیان متفاوت از سایر زبان­ها بجز عبری است. این تفاوت ناشی از بازنمائی ذهنی اعداد نبوده و باید علت را در سطح دیگری از پردازشها دنبال نمود.

Title: اثر بخشي نقاشي‌درماني بر کاهش رفتارهاي پرخاشگرانه دانش آموزان پسر دارای عقب‌ماندگی ذهني خفيف تا متوسط

http://jrrs.mui.ac.ir/index.php/jrrs/article/view/2028

مقدمه: رفتارهاي پرخاشگرانه، مشکلي متداول ميان کودکان عقب‌مانده‌ي ذهني است و يکي از روش‌هاي پيشنهاد شده، جهت درمان پرخاشگري، هنر درماني است. هدف اين پژوهش بررسي تأثير مداخلات نقاشي‌درماني برکاهش رفتارهاي پرخاشگرانه دانش‌آموزان پسر عقب‌مانده‌ي ذهني آموزش‌پذير مقطع ابتدايي بود. مواد و روش: 60 نفر از دانش‌آموزان پسرعقب مانده‌ي ذهني آموزش‌پذير مقطع ابتدايي، با روش نمونه‌گيري تصادفي خوشه‌اي انتخاب شدند. در اين پژوهش از روش آزمايشي با طرح پيش‌آزمون-  پس‌آزمون با گروه کنترل و گزينش تصادفي استفاده شد. براي گردآوري داده‌ها از پرسشنامه‌ي پرخاشگري کودکان کم توان ذهني (B. D. GH) فرم معلم استفاده گرديد. يافته ها: نتايج تحليل کوواريانس نشان داد که تفاوت بين دو گروه آزمايش و کنترل در پس‌آزمون با کسر اثر پيش‌آزمون در مولفه‌هاي پرخاشگري کلامي به خود (01/0P<، 58/20=F)، پرخاشگري غيركلامي به خود (01/0P<، 72/19=F)، پرخاشگري کلامي به ديگران (01/0P<، 53/22=F)، پرخاشگري غيركلامي به ديگران (01/0P<، 71/44=F) و نمره کل پرخاشگري (01/0P<، 85/56=F) معنادار است. نتيجه‌گيري: نتيجه اين پژوهش نشان داد، نقاشي‌درماني بطور معناداري باعث کاهش رفتارهاي پرخاشگرانه در دانش آموزان دارای عقب‌ماندگی ذهني مي‌شود، چون در نقاشي اين گروه از دانش آموزان فرصت مي‌يابند تا عواطف، احساسات و هيجانات متراکم شده و فشارهاي رواني حاصل از اين احساسات را از طريق نقاشي و به صورتي جامعه پسند نشان دهند، بنابراين کمتر پرخاشگري نشان مي‌دهند.

Title: راهبردهای آرام سازی نوزاد بر مبنای تجربه مادران ایرانی: یک مطالعه پدیدارشناختی

http://jrrs.mui.ac.ir/index.php/jrrs/article/view/1976

مقدمه:همچون هر تمدن دیگری،مادران ایرانی از دیرباز جهت آرام کردن نوزادان خود و مواجهه با مشکلات تنظیمی نوزاد از روش های سنتی و بومی مختلفی بهره می گرفتند. هدف این پژوهش عبارت بود از بررسی تجربه و نظر مادران ایرانی درباره روش آرام کردن نوزادانشان. مواد و روش ها: ملاک های ورود شرکت کنندگان در این پژوهش کیفی پدیدارشناختی عبارت بود از: تمایل  به حضور در پژوهش ، داشتن توانایی برقراری ارتباط ،  مادر بودن، داشتن سابقه مراقبت از حداقل یک نوزاد ،  ایرانی بودن و ملاک های خروج در این پژوهش عبارت بود از وجود مشکلات حافظه و اختلالات روانی. انتخاب شرکت کنندگان با استفاده از روش نمونه گیری هدفمند انجام شد و  به منظور گرداوری  اطلاعات 4 سوال در قالب مصاحبه عمیق و نیمه ساختار یافته )هر جلسه به مدت 30 تا 60 دقیقه) از شرکت کنندگان پرسیده شد. مصاحبه با شرکت کنندگان تا زمان اشباع اطلاعات با شرکت 21 نفرادامه یافت.در نهایت اطلاعات به دست آمده با استفاده از روش هفت مرحله ای کلایزی مورد تجزیه و تحلیل قرار  گرفت. یافته ها:داده های مربوط به تجربه های مادران منجر به ایجاد 8 تم شامل تحریکات وستیبولار، تحریکات لمسی، تحریکات شنیداری، تحریکات بینایی، تحریکات حرارتی، تحریکات حس عمقی، رفع نیاز های فیزیولوژیک و روش های  فرا-ماده و خرافی شده است. نتیجه گیری:یافته های حاصل از این مطالعه حاکی از وجود ارتباط بین  روش های سنتی و بومی که  مادران ایرانی جهت آرام کردن نوزادشان به کار می گیرند، با اصول علمی مبتنی بر نظریه یکپارچگی حسی است که می تواند با استفاده از پژوهش های مکمل کیفی و کمی مورد بررسی دقیق تر واقع شود.جهت بررسی تاثیر راهبردهایی که مادران ایرانی برای آرام سازی نوزادان خود استفاده می کنند، نیاز مطالعات متعدد کیفی و کمی وجود دارد.
